# Scalable discovery of homomeric protein–protein interactions from cross‐linking mass spectrometry data with CLAUDIO 2.0

**DOI:** 10.1002/pro.70732

**Published:** 2026-07-28

**Authors:** Tobias Löser, Alexander Röhl, Markus Baier, Andrei Lupas, Oliver Kohlbacher, Hadeer Elhabashy

**Affiliations:** ^1^ Applied Bioinformatics, Department of Computer Science University of Tübingen Tübingen Germany; ^2^ Department of Protein Evolution Max‐Planck‐Institute for Biology Tübingen Germany; ^3^ Institute for Bioinformatics and Medical Informatics University of Tübingen Tübingen Germany; ^4^ Institute for Translational Bioinformatics University Hospital Tübingen Tübingen Germany; ^5^ Artificial Intelligence in Protein Science University of Bayreuth Bayreuth Germany

**Keywords:** cross‐linking mass spectrometry, homomeric interaction

## Abstract

Cross‐linking mass spectrometry (XL‐MS) is a powerful biochemical approach for residue‐level characterization of protein structures and interactions under near‐native conditions. The growing scale of XL‐MS datasets demands scalable analysis pipelines that capture signals often overlooked in conventional workflows, including homomeric interactions. Here, we present CLAUDIO 2.0, a next‐generation framework for structural analysis of large‐scale XL‐MS data. CLAUDIO 2.0 identifies homomeric interactions using overlapping peptide sequences and structural evaluation. Our optimized workflow improves computational efficiency, enabling scalable analysis and expanding structural coverage. Applied to a human mitochondrial XL‐MS dataset, CLAUDIO 2.0 evaluates over 75% of cross‐links using available high‐confidence structural models, reduces runtime by over 95% (averaging 5 s per cross‐link) compared to its predecessor, and identifies 205 proteins with homomeric interaction signals. CLAUDIO 2.0 is freely available under the MIT License at (https://github.com/ElhabashyLab/CLAUDIO) and as a web server at (https://elhabashylab.org/claudio), providing an accessible platform for scalable structural proteomics.

## 1 INTRODUCTION

Cross‐linking mass spectrometry (XL‐MS) is a powerful method for probing protein structures and interaction networks at residue‐level resolution under near‐native conditions (Leitner et al., 2014; O'Reilly & Rappsilber, 2018; Sinz, 2018). By capturing spatial proximity between residues, XL‐MS provides distance constraints that complement established structural approaches such as cryo‐EM and computational modeling methods, including AlphaFold (Abramson et al., 2024; Evans et al., 2021; Jumper et al., 2021; Leitner et al., 2016). Emerging approaches, such as AlphaLink and AF3x, further demonstrate that integrating XL‐MS data as distance restraints or explicit cross‐links, respectively, into deep learning frameworks can improve the accuracy of protein structure and complex prediction (Gilep et al., 2024; Stahl et al., 2023).

The rapid growth of XL‐MS datasets demands computational tools that enable automated, scalable, and structure‐aware analysis. Existing tools such as Xlink Analyzer (Kosinski et al., 2015), Xwalk (Kahraman et al., 2011), and TopoLink (Ferrari et al., 2019) support structural interpretation but require substantial manual setup and do not scale to proteome‐wide studies. Beyond automation and scalability, XL‐MS datasets contain latent information, including evidence of homomeric interactions that are often overlooked or misinterpreted. In fact, XL‐MS is inherently biased toward abundant proteins, which are frequently enriched in homomeric complexes. Because interacting subunits share identical sequences, cross‐links within homomeric assemblies are often misclassified as intra‐protein cross‐links or discarded as false positives when they exceed distance thresholds in monomeric structures. As a result, bona fide inter‐chain restraints in homomeric assemblies can be obscured. Reliable identification of homomeric interactions from XL‐MS data therefore requires dedicated computational methods.

To address these limitations, we developed CLAUDIO (Cross‐Linking Analysis Using Distances and Overlaps), an integrated pipeline for structural analysis of cross‐linking data (Röhl et al., 2024). CLAUDIO integrates two complementary strategies to detect homomeric interactions. First, we introduced a sequence‐based analytical strategy based on overlapping peptide sequences (OPS). This approach exploits a simple yet rigorous principle: two cross‐linked peptides that share an identical sequence region cannot originate from the same polypeptide chain unless that region is repeated. Sequence overlap therefore provides direct, structure‐independent evidence for inter‐chain cross‐links within homomeric assemblies. Second, we further evaluated intra‐chain cross‐links structurally and reinterpreted those exceeding accepted distance thresholds in monomeric models as potential inter‐chain restraints. By combining sequence‐based inference with structural validation, CLAUDIO enables systematic and scalable identification of homomeric interactions from XL‐MS data. Previous applications demonstrate the scalability and utility of CLAUDIO in large‐scale studies. For example, Michael et al. used CLAUDIO to analyze over 41,000 cross‐linked peptides across 915 protein–protein interactions at a false discovery rate below 5% (Michael et al., 2024). Similarly, Amaral et al. mapped more than 7800 cross‐links onto 1465 protein structures from over 14,000 peptide pairs (Amaral et al., 2024). These studies highlight the applicability of CLAUDIO to large and complex XL‐MS datasets.

Here, we present CLAUDIO 2.0, a next‐generation framework that substantially accelerates XL‐MS data analysis through optimized parallelization strategies, vectorized computations, and efficient handling of structural data. Beyond its enhanced performance, CLAUDIO 2.0 is also available as a web‐based platform, providing an intuitive and accessible interface that allows users to perform large‐scale, structure‐aware XL‐MS analyses without the need for local installation or advanced computational expertise. Together, these improvements establish CLAUDIO 2.0 as a scalable, user‐friendly solution for comprehensive interpretation of cross‐linking datasets and systematic detection of homomeric interactions.

## 2 MATERIALS AND METHODS

CLAUDIO 2.0 is a Python package designed for scalable structural analysis of cross‐linking mass spectrometry (XL‐MS) data and the identification of homomeric interactions. It is freely available as an open‐source GitHub repository (https://github.com/ElhabashyLab/CLAUDIO) and as a web application (https://elhabashylab.org/claudio/). Building on its predecessor release, CLAUDIO 2.0 offers substantially improved computational efficiency and coverage through several methodological advances. These include parallelized and vectorized workflows, optimized handling of structural models with regular updates, local caching of structural databases to reduce repeated API calls, and additional technical enhancements that streamline processing and analysis. Table 1 summarizes the key methodological improvements between CLAUDIO 1.0 and 2.0.

**TABLE 1:** Key methodological improvements in CLAUDIO 2.0 compared to CLAUDIO 1.0.

Feature	CLAUDIO 1.0	CLAUDIO 2.0
Workflow efficiency	Sequential processing	Vectorized and parallelized workflow
NMR structure handling	Full NMR ensemble processed	Single representative model used per structure
PDB structure retrieval	API‐based retrieval	Local cached PDB database (~60 GB)
Database updates	Manual	Automated updates (default every 14 days)
AlphaFold database integration	AFDB version 1 (365,000 structure models)	AFDB version 6 (200 M+ structure models)
SWISS‐MODEL querying	Single‐protein per API call	Batch queries (250 proteins per API call)
Web server availability	Not available	User‐friendly web interface
Scalability	Limited for large datasets	Designed for proteome‐scale datasets
Homomeric interaction detection	Flags overlapping peptide sequences and out‐of‐range cross‐links	Explicit prediction of proteins with potential homomeric interaction signals

### 2.1 CLAUDIO 2.0 workflow

**FIGURE 1:**
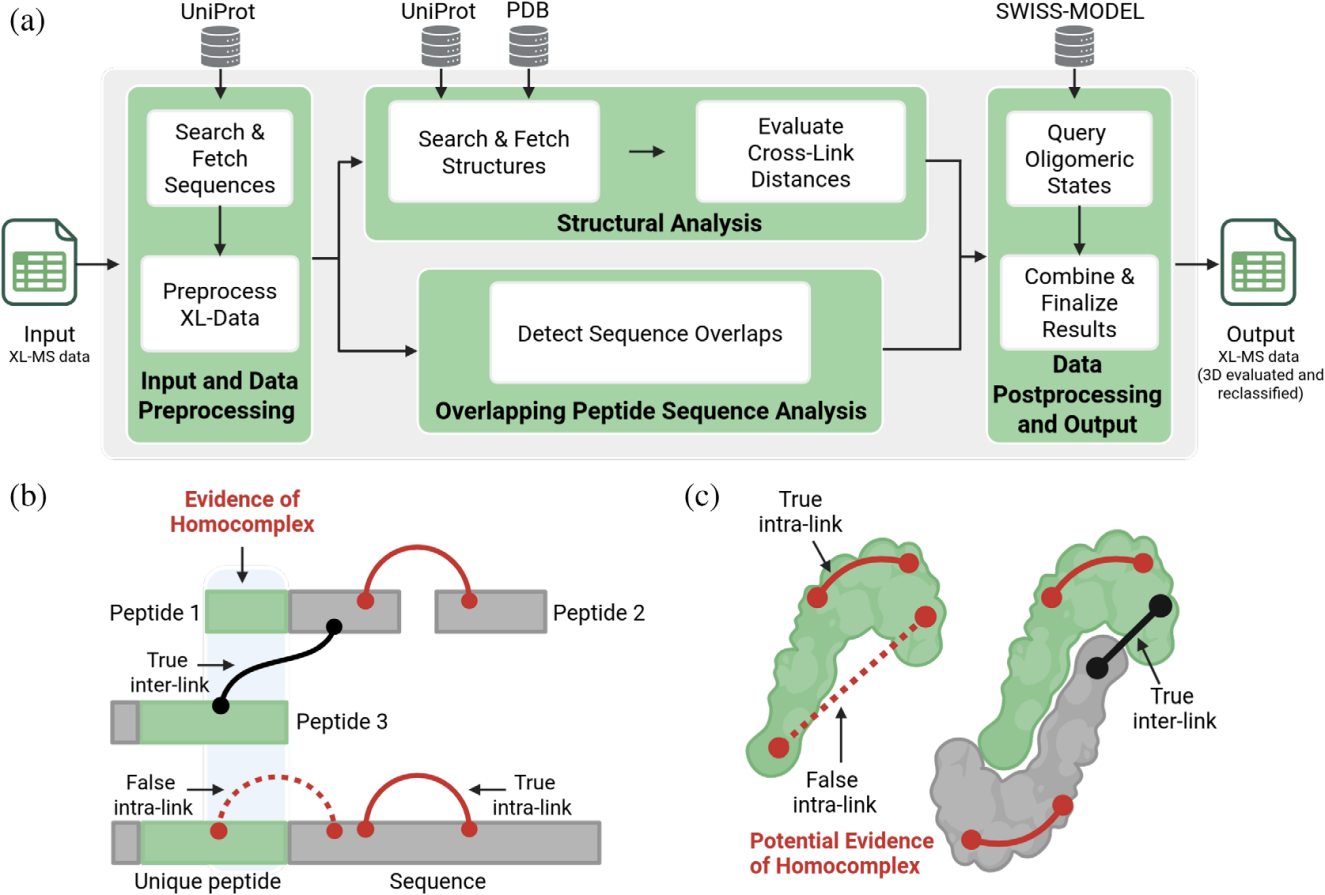
Cross‐link analysis in CLAUDIO 2.0. (a) Typical workflow of CLAUDIO 2.0, illustrating the four main modules and their parallel performance. (b) Schematic of the Overlapping Peptide Sequence (OPS) principle for detecting homomeric interactions. Peptide pairs sharing a sequence region (pale green) that is not repeated in the protein sequence (gray) indicate proximity of identical chains or potential homomeric interactions. These are illustrated as either false intra‐links (dashed red) or true inter‐links (black). Cross‐links between peptide pairs lacking OPS are classified as putative true inter‐links (red) pending structural validation. (c) Structural distance evaluation of inter‐ and intra‐links. Cross‐links within the distance threshold (solid red line) are accepted as intra‐protein links, while those exceeding the threshold (dashed red line) may indicate false positives when mapped onto a monomer. These cross‐links can be validated as inter‐links in the context of homomeric interaction (black).

The CLAUDIO 2.0 workflow is organized into four main modules: (I) Input and Data Preprocessing, (II) Structural Analysis, (III) Overlapping Peptide Sequence (OPS) Analysis, and (IV) Data Postprocessing and Output. A schematic overview of the typical CLAUDIO 2.0 pipeline is shown in Figure 1a. Modules II and III can be executed independently once the input data are preprocessed, while Module IV integrates results from the preceding modules to generate the final output. The following provides a brief description of each module. For detailed algorithmic steps and parameter settings, we refer readers to Röhl et al., 2024, supplementary material S1, and the GitHub repository documentation.

#### 2.1.1 Input and data preprocessing module

CLAUDIO 2.0 accepts cross‐linking mass spectrometry (XL‐MS) data in CSV format, containing essential information such as UniProt IDs, peptide sequences, and residue positions. Protein sequences are retrieved from UniProtKB using the provided UniProt IDs. CLAUDIO then verifies and corrects any discrepancies in residue numbering or amino acid identity between the reported positions and the actual sequences via peptide‐to‐protein alignment. This alignment also enables the identification of repeated peptide instances, all of which are considered in the downstream structural evaluation. Additionally, users can define valid cross‐linkable residues, specifying permissible amino acids, atom types, and distance constraints for the used chemical cross‐linker.

#### 2.1.2 Structural analysis module

The structural analysis module evaluates the spatial feasibility of identified cross‐links (Figure 1c) by retrieving corresponding protein structures. Protein structures are obtained by aligning UniProt sequences (Consortium U, 2015) against the RCSB Protein Data Bank (PDB) (Burley et al., 2023). These structures are then used to compute distances between cross‐linked residues. For NMR‐derived structures, CLAUDIO 2.0 considers only a single representative model for distance evaluation, improving computational efficiency compared to the previous version, which processed the full ensemble. To minimize retrieval time resulting from API calls for structure, CLAUDIO 2.0 uses a locally cached PDB database (≈60 GB) that can be automatically updated at user‐defined intervals (default: 14 days). In addition, this release automates the download and updating of both the SIFTS and pdbaa databases. When experimental structures are unavailable for intra‐protein cross‐links, computational models from the AlphaFold Protein Structure Database (AFDB) are retrieved (Varadi et al., 2022; Varadi et al., 2024). Version 6 of AFDB, containing over 200 million models, is used in CLAUDIO 2.0, greatly expanding coverage compared to Version 1 in the previous release. Inter‐protein cross‐links are analyzed only when a common PDB structure contains both cross‐linked proteins. If multiple chains are present in a PDB entry, all possible chain permutations are treated as separate data points, which can increase both dataset size and runtime. Finally, the module computes Euclidean and topological distances between cross‐linked residues using TopoLink (Ferrari et al., 2019). Here, the topological distance represents the minimum surface‐accessible path required for a physical linker to connect reactive atoms without passing through protein atoms, thereby providing structural validation of the observed cross‐links.

#### 2.1.3 Overlapping peptide sequence analysis module

The Overlapping Peptide Sequence (OPS) module detects potential homomeric interactions by identifying overlapping peptide sequences in intra‐cross‐linked peptides. The underlying principle is that when two cross‐linked peptides share a sequence segment that is unique within the protein, they most likely originate from separate, identical chains, indicating proximity between identical chains (Figure 1b). However, peptides originating from repeated sequences within a single chain do not automatically signal homomeric interactions, since overlaps in these cases could arise from the same polypeptide chain. CLAUDIO aligns cross‐linked peptides to the protein sequence and flags overlaps and repeats, providing evidence for potential inter‐chain contacts.

#### 2.1.4 Data postprocessing and output module

The final module of CLAUDIO 2.0 integrates results from the structural analysis and OPS modules to provide a comprehensive classification of cross‐links. Same‐protein cross‐links are reclassified as inter‐links when supported by either peptide sequence overlap or structural evaluation outside the accepted distance range, which depends on the cross‐linker used. For DSS, we applied a topological distance range of 5–30 Å, reflecting its spacer length and accounting for side‐chain flexibility of the cross‐linked residues. This threshold can be adjusted by the user depending on the properties of the cross‐linker employed. To further validate these assignments, the module queries the SWISS‐MODEL repository for known multimeric states, confirming homo‐oligomeric arrangements by homology when available. CLAUDIO 2.0 can query the SWISS‐MODEL API in batches of up to 250 proteins per request, a significant improvement over the previous version, which processed one protein at a time, enhancing computational efficiency. Cross‐links lacking known structural evidence are flagged as potential new homo‐multimer candidates for further investigation. The module outputs a CSV file containing the original cross‐links, computed Euclidean and topological distances, and assigned cross‐link types. In addition, CLAUDIO 2.0 generates PyMOL sessions mapping all cross‐links onto their corresponding structures, along with summary figures including distance histograms, cross‐link type distributions, and structural statistics. A log file documents preprocessing steps such as corrected residue positions, removal of duplicates, and creation of new datapoints from repeated peptides.

### 2.2 Web server implementation

The CLAUDIO web server (https://elhabashylab.org/claudio) provides a user‐friendly platform for structural analysis of cross‐linking mass spectrometry (XL‐MS) data, eliminating the need for local installation or command‐line expertise. The front‐end interface is built with HTML5 and the Bootstrap framework (v5.3.3), while PHP (v8.3.6) manages backend logic and MySQL (v8.0.41) manages the underlying databases. The server runs a local installation of CLAUDIO 2.0 and supports scalable deployment and job queuing via SLURM. It is hosted on a secure system provided by the German Network for Bioinformatics Infrastructure (de.NBI), running Ubuntu 24.04 LTS (2024‐08‐13) with 28 VCPU cores and 512 GB RAM. CLAUDIO's databases are updated every 14 days, and the versions used for each analysis are logged with the results. Uploaded data are treated as confidential, stored only for the duration of analysis, and automatically deleted after 14 days. Users can search for completed jobs without logging in, and no personal information is collected or retained. The web server was rigorously validated using the benchmark dataset and cross‐checked against the standalone CLAUDIO 2.0 command‐line tool to ensure consistency of results. Automatic database updates ensure that users always access the most current structural and sequence information. This approach combines open‐source accessibility and regular maintenance to guarantee the sustainability, reproducibility, and long‐term usability of CLAUDIO.

### 2.3 Performance assessment

We assessed the performance of CLAUDIO 2.0 (latest release date: 23 March 2026) in comparison to CLAUDIO 1.0.1 (release date: 30.10.2024) using Python's cProfile and pStats profiling modules (Python 3.11). Both versions were evaluated on the same benchmark dataset and computational infrastructure to ensure a fair and reproducible comparison. As a benchmark, we used a previously reported cross‐linking mass spectrometry dataset generated with the disuccinimidyl suberate (DSS) linker on human mitochondria, comprising 5518 cross‐links at a false discovery rate (FDR) below 5% (Ryl et al., 2019). The dataset CSV file is available on the CLAUDIO GitHub repository. All analyses were performed on a standardized infrastructure running Ubuntu 24.04 LTS (2024‐08‐13), equipped with 28 vCPU cores and 512 GB RAM. To evaluate computational efficiency under realistic usage conditions, we compared the standalone CLAUDIO 2.0 implementation, as deployed on the web server, against CLAUDIO 1.0.1 executed on the same system. This setup ensures consistency and reproducibility across different modes of application. Performance was quantified using cumulative runtime, capturing total execution time including all nested and recursive function calls. We report cumulative runtime per run, as well as module‐level contributions and normalized runtime per cross‐link, enabling fine‐grained comparison between versions.

## 3 RESULTS

**FIGURE 2:**
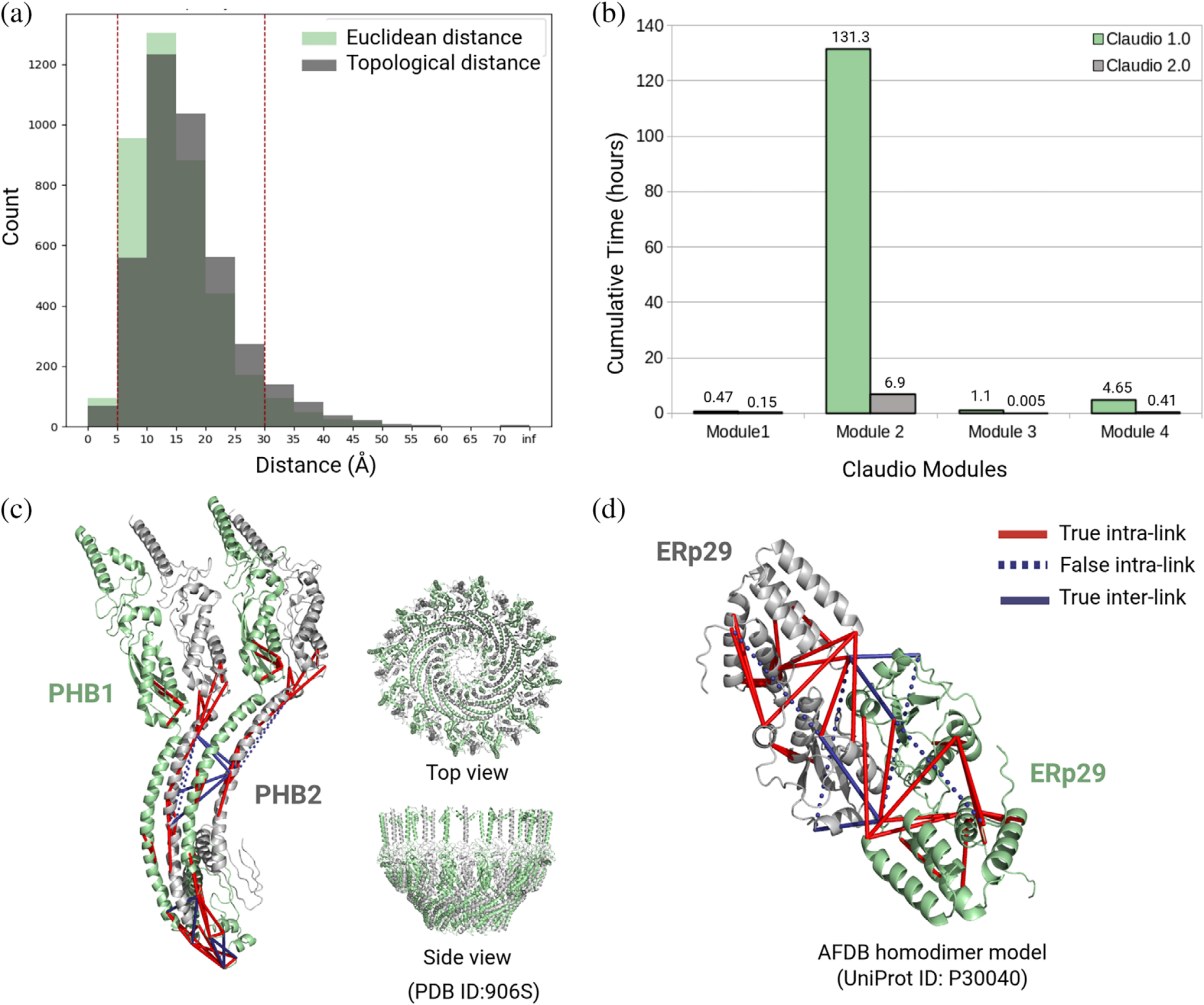
(a) Histogram of cross‐link distances in the human mitochondrial dataset evaluated by CLAUDIO 2.0. Euclidean distances are shown in pale green, topological distances in gray. Red dashed lines indicate the accepted distance range (5–30) Å. (b) Cumulative runtime across CLAUDIO 2.0 modules compared with CLAUDIO 1. Module 2 (Structural Analysis) is a bottleneck in CLAUDIO 1, whereas CLAUDIO 2.0 achieves a substantial reduction in runtime and improved computational efficiency due to optimized implementation. (c) Top and side views of the human prohibitin complex (PDB ID: 9O6S). Cross‐links are shown on a tetramer comprising two PHB1 subunits (pale green, UniProt ID: aP35232) and two PHB2 subunits (gray, UniProt ID: Q99623). Red lines indicate intra‐chain cross‐links within the accepted distance range (5–30) Å, dashed blue lines represent false intra‐chain links exceeding 30 Å, and solid blue lines represent remapped inter‐chain cross‐links between identical subunits within a homomeric assembly. (d) Cross‐links mapped onto the AlphaFold homodimer model of ERp29 (AF‐0000000065885228‐v1; UniProt ID: P30040).

We applied CLAUDIO 2.0 to a previously reported human mitochondrial dataset (5518 cross‐links) generated by cross‐linking mass spectrometry (XL‐MS) using the non‐cleavable cross‐linker DSS. This benchmark dataset comprises, after pre‐processing, 5488 non‐redundant cross‐linked residue pairs across 790 proteins, identified at a false discovery rate (FDR) below 5%. Of these, 152 cross‐links (2.8%) correspond to inter‐protein interactions, while the majority (5336 cross‐links) represent intra‐protein cross‐links (Ryl et al., 2019). CLAUDIO 2.0 was able to structurally evaluate 4031 cross‐links (75.5% of the dataset), with the remaining cross‐links lacking high‐confidence structural information (Figure 2a). Of the evaluated cross‐links, 3940 satisfied the accepted distance range of 5–30 Å, indicating agreement with available structural models.

Among intra‐protein cross‐links, CLAUDIO 2.0 identified 537 cross‐links across 205 proteins as putative homomeric interaction signals. Cross‐links involving identical or overlapping peptide sequences accounted for 173 cross‐links across 88 proteins. For 59 of these 88 proteins (~67%), homologous structures annotated in the SWISS‐MODEL Repository (Bienert et al., 2017; Kopp & Schwede, 2004) indicate known or predicted homomeric assemblies. The structural analysis module flagged the remaining 364 out‐of‐range cross‐links across 157 proteins as potential homomeric interaction signals. Subsequent searches against the SWISS‐MODEL Repository identified homologous proteins involved in homomeric assemblies for 61 proteins (~39%) within this set. This class of evidence is inherently weaker, as it may include propagated false positives or reflect structural inaccuracies in the underlying models; nevertheless, these candidates still represent plausible hypotheses for homomeric interactions. A subset of proteins was detected by both the OPS and structural analysis modules as being involved in homomeric interactions. By integrating these complementary sources of evidence, CLAUDIO 2.0 predicts that 205 human mitochondrial proteins exhibit homomeric interaction signals. Of these, a non‐redundant set of 77 proteins is further supported by homologous structures in the SWISS‐MODEL Repository, which indicate homomeric assemblies. The remaining 128 proteins represent potential homomeric interactions that warrant further literature mining and experimental validation.

As an illustrative example, Prohibitin 1 (PHB1) and Prohibitin 2 (PHB2), members of the SPFH protein family, form the core of the mitochondrial prohibitin complex, which assembles into large hetero‐oligomeric supercomplexes (~1 MDa) (Coates et al., 2001; Wei et al., 2017; Yoshinaka et al., 2019). Embedded in the inner mitochondrial membrane, this complex is essential for maintaining cristae architecture, stabilizing respiratory chain complexes, and regulating mitochondrial proteostasis. Within the dataset, CLAUDIO 2.0 identified 17 intra‐protein cross‐links for PHB1, 20 for PHB2, and 23 inter‐protein cross‐links between the two proteins. For PHB1, three intra‐links involved identical or overlapping peptide sequences, and four exceeded the 30 Å distance threshold when mapped onto monomeric structures. For PHB2, four intra‐links showed identical or overlapping peptide regions (Figure 2c). Mapping these cross‐links onto the recently resolved structure of the human prohibitin complex (PDB ID: 9O6S) confirms that the inferred restraints are inter‐chain links consistent with the expected distance constraints within the homomeric assembly, thereby validating the predictive capability of the approach. As another example, the endoplasmic reticulum resident protein 29 (ERp29; UniProt ID: P30040), involved in processing and folding of secretory proteins within the endoplasmic reticulum (Ferrari et al., 1998), exhibited 21 intra‐protein cross‐links. Among these, one cross‐link showed overlapping peptide sequences, and three exceeded the accepted distance threshold when mapped onto the monomeric structure (Figure 2d). Consistent with these observations, ERp29 is known to form a homodimer under physiological conditions (Rainey‐Barger et al., 2007). Mapping the flagged cross‐links onto an AFDB‐predicted homodimer model (Varadi et al., 2022; Varadi et al., 2024) showed that three cross‐links satisfy expected distance constraints, supporting their reinterpretation as inter‐chain restraints and providing structural evidence for homodimerization.

On the computational side, CLAUDIO 2.0 also demonstrates improved robustness. Designed for large‐scale datasets, the pipeline achieves an average runtime of ~5 s per cross‐link, resulting in a total runtime of 7 h 30 min 33 s for the benchmark dataset (Figure 2b). In comparison, CLAUDIO 1.0 required ~90 s per cross‐link and was frequently constrained by structural analysis bottlenecks, particularly when processing NMR ensembles with multiple models, which could stall evaluation.

CLAUDIO 2.0 addresses this limitation by selecting representative structures for NMR ensembles rather than evaluating all models. Benchmarking (Figure 2b) shows that structural evaluation remains the dominant computational cost in both versions; however, vectorized operations, parallelization, and the use of local structural databases (replacing API‐based retrieval in CLAUDIO 1.0) substantially improve performance. Collectively, these optimizations reduce total runtime by 95%, enabling scalable analysis of large XL‐MS datasets. In addition, CLAUDIO 2.0 is available as a plug‐and‐play web interface, providing broad accessibility without requiring local installation or specialized computational expertise.

## 4 DISCUSSION

Cross‐linking mass spectrometry (XL‐MS) has emerged as a powerful approach for probing protein–protein interactions under near‐native conditions. However, cross‐links report on spatial proximity rather than direct biochemical interactions. In densely packed environments or multimeric assemblies, this can lead to ambiguity, as cross‐links may arise between adjacent subunits without reflecting stable or specific interfaces. At the same time, XL‐MS datasets contain additional layers of information that remain underexplored. In particular, cross‐linking patterns can provide insights into conformational changes and dynamic aspects of protein interactions. Systematic approaches that mine such information could further expand the utility of XL‐MS for studying structural plasticity and interaction dynamics.

An important yet often underappreciated feature of XL‐MS datasets is their bias toward abundant proteins. Mass spectrometry preferentially detects peptides from highly abundant species, which are often homomeric proteins existing in multiple identical copies. As a result, XL‐MS data are naturally enriched for homomeric interactions. The OPS analysis implemented in CLAUDIO is particularly powerful for detecting such interactions, as its sequence‐based, structure‐independent nature provides a largely assumption‐free criterion that captures signals often missed by distance‐based approaches. Despite its strengths, this approach has limitations. Overlapping peptides do not provide information about oligomerization states and therefore cannot distinguish between dimers, trimers, or higher‐order assemblies. Peptides derived from repeated sequence regions within a single polypeptide chain can also produce apparent overlaps that do not reflect true homomeric interactions. CLAUDIO identifies and reports peptides originating from repeated regions.

Integrating cross‐linking data with structural information further enhances interpretability. Mapping cross‐links onto structural models—whether derived from experimental structures, homology modeling, or prediction frameworks such as AlphaFold—facilitates discrimination between intra‐chain constraints and plausible inter‐chain contacts. However, this integration is subject to limitations: discrepancies between cross‐link distances and structural models may arise from incomplete or inaccurate models, mismatched oligomeric states, or alternative conformations. The simplification of NMR ensemble processing to a single representative model, for instance, improves scalability but may also cause cross‐links compatible with alternative conformers to be incorrectly flagged as distance violations; this trade‐off is acceptable for proteome‐wide screening but warrants careful consideration in targeted studies of conformationally heterogeneous proteins. Such inconsistencies may also arise from false‐positive cross‐links, for example due to gas‐phase adduct formation in mass spectrometry, which can produce misleading peaks or spurious associations. The absence of high‐confidence structural models limits the ability to validate the detected crosslinks or contextualize new homomeric interactions.

The rapidly increasing scale of XL‐MS experiments presents an additional challenge. Modern studies can generate tens of thousands of cross‐links in a single experiment, requiring computational frameworks that are both reliable and highly efficient. CLAUDIO meets this need through optimized, parallelized implementation, enabling high‐throughput analysis of large‐scale datasets without compromising accuracy. Equally important is accessibility: many computational tools require substantial technical expertise, limiting their utility for experimentalists. CLAUDIO overcomes this barrier through a user‐friendly, web‐based interface and a plug‐and‐play workflow, allowing researchers to customize runs and analyze their data efficiently without computational background.

By combining conceptual simplicity, scalable implementation, and an accessible web interface, CLAUDIO 2.0 is well‐positioned to meet the demands of increasingly large in‐cell and in situ XL‐MS datasets, where homomeric signals are expected to be especially prevalent.

## AUTHOR CONTRIBUTIONS

**Andrei Lupas:** Supervision; resources; funding acquisition; project administration; writing – review and editing. **Hadeer Elhabashy:** Conceptualization; writing – original draft; writing – review and editing; supervision; investigation; software; visualization; validation; methodology; project administration; formal analysis. **Alexander Röhl:** Software; writing – review and editing; writing – original draft; data curation; investigation; formal analysis; visualization; validation. **Oliver Kohlbacher:** Supervision; resources; funding acquisition; project administration; writing – review and editing. **Tobias Löser:** Software; writing – original draft; formal analysis; data curation; investigation; visualization; validation; writing – review and editing. **Markus Baier:** Software; writing – review and editing.

## FUNDING INFORMATION

This study was supported by institutional funds from the Max Planck Institute for Biology Tübingen and the University of Tübingen.

## CONFLICT OF INTEREST STATEMENT

The authors declare that they have no conflicts of interest.

## Supporting information


**DATA S1.** For detailed documentation, we refer readers to the DataS1 of Röhl et al., 2024, and the GitHub repository https://github.com/ElhabashyLab/CLAUDIO. Additionally, the benchmark dataset together with outputs generated using CLAUDIO 1.0 and CLAUDIO 2.0 are provided as Data S1.

## Data Availability

The data that supports the findings of this study are available in the supplementary material of this article.

## References

[pro70732-bib-0001] Abramson J , Adler J , Dunger J , Evans R , Green T , Pritzel A , et al. Accurate structure prediction of biomolecular interactions with AlphaFold 3. Nature. 2024;630(8016):493–500. 38718835 10.1038/s41586-024-07487-w PMC11168924

[pro70732-bib-0002] Amaral BC , Michael AR , Brodie NI , Crowder DA , Eiriksson KH , Schriemer DC . Click‐linking: a cell‐compatible protein crosslinking method based on click chemistry. bioRxiv. 2024;2024. 10.1038/s41467-025-64888-9 PMC12592537 41198667

[pro70732-bib-0003] Bienert S , Waterhouse A , de Beer TA , Tauriello G , Studer G , Bordoli L , et al. The SWISS‐MODEL repository: new features and functionality. Nucleic Acids Res. 2017;45(D1):D313–D319. 27899672 10.1093/nar/gkw1132 PMC5210589

[pro70732-bib-0004] Burley SK , Bhikadiya C , Bi C , Bittrich S , Chao H , Chen L , et al. RCSB Protein Data Bank (RCSB. org): delivery of experimentally‐determined PDB structures alongside one million computed structure models of proteins from artificial intelligence/machine learning. Nucleic Acids Res. 2023;51(D1):D488–D508. 36420884 10.1093/nar/gkac1077 PMC9825554

[pro70732-bib-0005] Coates P , Nenutil R , McGregor A , Picksley S , Crouch D , Hall P , et al. Mammalian prohibitin proteins respond to mitochondrial stress and decrease during cellular senescence. Exp Cell Res. 2001;265(2):262–273. 11302691 10.1006/excr.2001.5166

[pro70732-bib-0006] Consortium U . UniProt: a hub for protein information. Nucleic Acids Res. 2015;43(D1):D204–D212. 25348405 10.1093/nar/gku989 PMC4384041

[pro70732-bib-0007] Evans R , O'Neill M , Pritzel A , Antropova N , Senior A , Green T , et al. Protein complex prediction with AlphaFold‐Multimer. bioRxiv. 2021;2021.

[pro70732-bib-0008] Ferrari AJ , Clasen MA , Kurt L , Carvalho PC , Gozzo FC , Martínez L . TopoLink: evaluation of structural models using chemical crosslinking distance constraints. Bioinformatics. 2019;35(17):3169–3170. 30629147 10.1093/bioinformatics/btz014

[pro70732-bib-0009] Ferrari DM , Nguyen Van P , Kratzin HD , Söling HD . ERp28, a human endoplasmic‐reticulum‐lumenal protein, is a member of the protein disulfide isomerase family but lacks a CXXC thioredoxin‐box motif. Eur J Biochem. 1998;255(3):570–579. 9738895 10.1046/j.1432-1327.1998.2550570.x

[pro70732-bib-0010] Gilep K , Obarska‐Kosinska A , Kosinski J . Improving AlphaFold 3 structural modeling by incorporating explicit crosslinks. bioRxiv. 2024;2024.

[pro70732-bib-0011] Jumper J , Evans R , Pritzel A , Green T , Figurnov M , Ronneberger O , et al. Highly accurate protein structure prediction with AlphaFold. Nature. 2021;596(7873):583–589. 34265844 10.1038/s41586-021-03819-2 PMC8371605

[pro70732-bib-0012] Kahraman A , Malmström L , Aebersold R . Xwalk: computing and visualizing distances in cross‐linking experiments. Bioinformatics. 2011;27(15):2163–2164. 21666267 10.1093/bioinformatics/btr348 PMC3137222

[pro70732-bib-0013] Kopp J , Schwede T . The SWISS‐MODEL repository of annotated three‐dimensional protein structure homology models. Nucleic Acids Res. 2004;32(suppl_1):D230–D234. 14681401 10.1093/nar/gkh008 PMC308743

[pro70732-bib-0014] Kosinski J , von Appen A , Ori A , Karius K , Müller CW , Beck M . Xlink analyzer: software for analysis and visualization of cross‐linking data in the context of three‐dimensional structures. J Struct Biol. 2015;189(3):177–183. 25661704 10.1016/j.jsb.2015.01.014 PMC4359615

[pro70732-bib-0015] Leitner A , Faini M , Stengel F , Aebersold R . Crosslinking and mass spectrometry: an integrated technology to understand the structure and function of molecular machines. Trends Biochem Sci. 2016;41(1):20–32. 26654279 10.1016/j.tibs.2015.10.008

[pro70732-bib-0016] Leitner A , Joachimiak LA , Unverdorben P , Walzthoeni T , Frydman J , Förster F , et al. Chemical cross‐linking/mass spectrometry targeting acidic residues in proteins and protein complexes. Proc Natl Acad Sci. 2014;111(26):9455–9460. 24938783 10.1073/pnas.1320298111 PMC4084482

[pro70732-bib-0017] Michael AR , Amaral BC , Ball KL , Eiriksson KH , Schriemer DC . Cell fixation improves performance of in situ crosslinking mass spectrometry while preserving cellular ultrastructure. Nat Commun. 2024;15(1):8537. 39358380 10.1038/s41467-024-52844-y PMC11447256

[pro70732-bib-0018] O'Reilly FJ , Rappsilber J . Cross‐linking mass spectrometry: methods and applications in structural, molecular and systems biology. Nat Struct Mol Biol. 2018;25(11):1000–1008. 30374081 10.1038/s41594-018-0147-0

[pro70732-bib-0019] Rainey‐Barger EK , Mkrtchian S , Tsai B . Dimerization of ERp29, a PDI‐like protein, is essential for its diverse functions. Mol Biol Cell. 2007;18(4):1253–1260. 17267685 10.1091/mbc.E06-11-1004 PMC1838973

[pro70732-bib-0020] Röhl A , Netz E , Kohlbacher O , Elhabashy H . CLAUDIO: automated structural analysis of cross‐linking data. Bioinformatics. 2024;40(4):btae146. 10.1093/bioinformatics/btae146 38498849 PMC10994719

[pro70732-bib-0021] Ryl PS , Bohlke‐Schneider M , Lenz S , Fischer L , Budzinski L , Stuiver M , et al. In situ structural restraints from cross‐linking mass spectrometry in human mitochondria. J Proteome Res. 2019;19(1):327–336. 31746214 10.1021/acs.jproteome.9b00541 PMC7010328

[pro70732-bib-0022] Sinz A . Cross‐linking/mass spectrometry for studying protein structures and protein–protein interactions: where are we now and where should we go from here? Angew Chem Int Ed. 2018;57(22):6390–6396. 10.1002/anie.201709559 29334167

[pro70732-bib-0023] Stahl K , Graziadei A , Dau T , Brock O , Rappsilber J . Protein structure prediction with in‐cell photo‐crosslinking mass spectrometry and deep learning. Nat Biotechnol. 2023;41(12):1810–1819. 36941363 10.1038/s41587-023-01704-z PMC10713450

[pro70732-bib-0024] Varadi M , Anyango S , Deshpande M , Nair S , Natassia C , Yordanova G , et al. AlphaFold protein structure database: massively expanding the structural coverage of protein‐sequence space with high‐accuracy models. Nucleic Acids Res. 2022;50(D1):D439–D444. 34791371 10.1093/nar/gkab1061 PMC8728224

[pro70732-bib-0025] Varadi M , Bertoni D , Magana P , Paramval U , Pidruchna I , Radhakrishnan M , et al. AlphaFold protein structure database in 2024: providing structure coverage for over 214 million protein sequences. Nucleic Acids Res. 2024;52(D1):D368–D375. 37933859 10.1093/nar/gkad1011 PMC10767828

[pro70732-bib-0026] Wei Y , Chiang WC , Sumpter R , Mishra P , Levine B . Prohibitin 2 is an inner mitochondrial membrane mitophagy receptor. Cell. 2017;168(1):224–238. 28017329 10.1016/j.cell.2016.11.042 PMC5235968

[pro70732-bib-0027] Yoshinaka T , Kosako H , Yoshizumi T , Furukawa R , Hirano Y , Kuge O , et al. Structural basis of mitochondrial scaffolds by prohibitin complexes: insight into a role of the coiled‐coil region. IScience. 2019;19:1065–1078. 31522117 10.1016/j.isci.2019.08.056 PMC6745515

